# Connecting Different Data Sources to Assess the Interconnections between Biosecurity, Health, Welfare, and Performance in Commercial Pig Farms in Great Britain

**DOI:** 10.3389/fvets.2018.00041

**Published:** 2018-03-06

**Authors:** Fanny Pandolfi, Sandra A. Edwards, Dominiek Maes, Ilias Kyriazakis

**Affiliations:** ^1^School of Agriculture Food and Rural Development, Newcastle University, Newcastle upon Tyne, United Kingdom; ^2^Faculty of Veterinary Medicine, Ghent University, Merelbeke, Belgium

**Keywords:** biosecurity, databases, health, lameness, pig, production performance, tail biting, welfare

## Abstract

This study aimed to provide an overview of the interconnections between biosecurity, health, welfare, and performance in commercial pig farms in Great Britain. We collected on-farm data about the level of biosecurity and animal performance in 40 fattening pig farms and 28 breeding pig farms between 2015 and 2016. We identified interconnections between these data, slaughterhouse health indicators, and welfare indicator records in fattening pig farms. After achieving the connections between databases, a secondary data analysis was performed to assess the interconnections between biosecurity, health, welfare, and performance using correlation analysis, principal component analysis, and hierarchical clustering. Although we could connect the different data sources the final sample size was limited, suggesting room for improvement in database connection to conduct secondary data analyses. The farm biosecurity scores ranged from 40 to 90 out of 100, with internal biosecurity scores being lower than external biosecurity scores. Our analysis suggested several interconnections between health, welfare, and performance. The initial correlation analysis showed that the prevalence of lameness and severe tail lesions was associated with the prevalence of enzootic pneumonia-like lesions and pyaemia, and the prevalence of severe body marks was associated with several disease indicators, including peritonitis and milk spots (*r* > 0.3; *P* < 0.05). Higher average daily weight gain (ADG) was associated with lower prevalence of pleurisy (*r* > 0.3; *P* < 0.05), but no connection was identified between mortality and health indicators. A subsequent cluster analysis enabled identification of patterns which considered concurrently indicators of health, welfare, and performance. Farms from cluster 1 had lower biosecurity scores, lower ADG, and higher prevalence of several disease and welfare indicators. Farms from cluster 2 had higher biosecurity scores than cluster 1, but a higher prevalence of pigs requiring hospitalization and lameness which confirmed the correlation between biosecurity and the prevalence of pigs requiring hospitalization (*r* > 0.3; *P* < 0.05). Farms from cluster 3 had higher biosecurity, higher ADG, and lower prevalence for some disease and welfare indicators. The study suggests a smaller impact of biosecurity on issues such as mortality, prevalence of lameness, and pig requiring hospitalization. The correlations and the identified clusters suggested the importance of animal welfare for the pig industry.

## Introduction

Increasing productivity of intensive farming systems and the production diseases which are connected to this make it necessary to better understand the interconnections between animal health, welfare and productivity. Food scares have raised the interest in farm animal welfare and health among society and caused policy makers not to only focus on farm productivity but also increase their concern about the ethical treatment of animals.

To assess health, welfare, and performance in commercial pig farms, data need to be collected. Collecting primary data can be relatively expensive and time consuming. In the livestock sector, many data are collected by both public and private sector bodies for purposes other than research (1, 2). These data may represent an opportunity to conduct secondary data analyses and offer a cost-effective approach to address research questions (3–5), including ones relating to animal health and welfare. Access to large sample numbers, recorded over long periods of time, time saving and lower cost are generally reported as some of the advantages of secondary data analysis (2, 3, 5). These data can also be used to complete findings from a primary study (5). At the same time, several disadvantages have also been reported, including poor control of the studied populations and measures (3). When using several data sources, the ability to connect the different databases will determine the quality of the study and can greatly affect the sample available to conduct the analysis. With the objective of using the available resources in a cost-effective and sustainable way, the potential for connection between different data sources related to pig health, welfare, and performance needs to be assessed. This study addressed the challenge of collecting and connecting different databases and how secondary data analysis could result in a better understanding of the challenges of the pig industry. From a more holistic perspective, this also illustrates the potential opportunity represented by secondary data analysis in research and highlights some practical limitations and possible improvements for collecting, connecting and analyzing large datasets to perform quality research.

Large datasets exist within the pig industry which document the prevalence of indicators of health and welfare collected on-farm or at the abattoir (6–8). The British Pig Health Scheme (BPHS) is one of the most developed national programs for post mortem assessment of pig health and the British pig industry was the first to conduct an assessment of pig welfare at national level through the Real Welfare project, with no equivalent of large-scale on-farm assessment available elsewhere. A few studies have investigated the connection between different abattoir data and carcass weight (9–11), but the connections between these data and extensive on-farm data have seldom been made. Associations between pig health, welfare, and performance have been identified in many studies (11–13). For example, tail lesion prevalence, which is considered as one of the most important welfare indicators, has been connected to sneezing frequency (14), acute phase protein titers and abscesses (15), and lung lesions (16). While biosecurity, health, welfare, and performance have been well studied individually, they have seldom been assessed in an integrated manner, and their connections need to be further explored. Moreover, connections between biosecurity and welfare in pig farms are still lacking in the literature.

This study aimed at identifying possible connections between on-farm data and large-scale industry databases holding complementary information and aimed at understanding if better welfare and biosecurity are connected to better health and performance. Our objective was to define hypotheses regarding the connection between biosecurity, health, welfare, and performance that could be challenged by using larger samples and other methodologies.

## Materials and Methods

The identification of interconnections between biosecurity, health, welfare, and performance in commercial pig farms was achieved by connecting data collected for different purposes over the same time period. Initially, a survey was conducted to collect on-farm data about animal performance and assess the level of biosecurity in commercial breeding and fattening pig farms in Great Britain. Subsequently, we identified the connections between these data collected on-farm and two different large-scale industry databases holding information about commercial pig farms in Great Britain: indicators of health and welfare collected by the Agricultural and Horticultural Development Board (AHDB) Pork for the BPHS and Real Welfare Scheme. After achieving the connections between different databases, we conducted a secondary data analysis to assess the interconnections between biosecurity, health, welfare, and performance in commercial pig farms in Great Britain.

All farmers provided a written consent to participate to the study, farmer anonymity was preserved, and none of the identifying information was included in the manuscript.

### Sampling

#### Farm Classification

A list of the county parish holding (CPH) number and the number of breeding pigs and fattening pigs of all pig farms in Great Britain were obtained from the Animal and Plant Health Agency and the Scottish Government Rural and Environment Science and Analytical Services (RESAS) in 2014. The most recent data communicated for fattening pigs allowing us to perform farm classification based on the same year for the three countries (England, Wales, and Scotland) were from 2010. The population figures (number of breeding pigs and number of other pigs) of all pig farms in these three countries were used to stratify the population similarly to the EUROSTAT classification (17).

The whole population of fattening pig farms was classified into four different groups according to herd size: group 1: small fatteners (no breeding pigs and less than 10 other pigs), group 2: large fatteners (no breeding pigs; at least 400 other pigs), group 3: large breeder-fatteners (at least 400 other pigs and 100 breeding pigs), and group 4: other farms fitting none of these definitions. In this analysis, breeding pigs were defined according to available data as sows, gilts, suckled or dry sows or dry sows kept for further breeding and gilts of 50 kg and over expected to be used or sold for breeding. The other pigs were defined as all fattening pigs over 20 kg including barren sows. The whole population of breeding pig farms in England, Wales and Scotland was used to classify the farms into two groups as follows: group A: specialized breeders with no fattening pigs over 20 kg and group B: breeding-fattening herds with at least one fattening pig over 20 kg in the herd.

#### Sampling

We obtained two convenience samples: one with fattening pig farms (specialized fatteners and breeder-fatteners) and the other with breeding farms (specialized breeders or breeder-fatteners), with some overlap (breeding-fattening farms were included in both categories of farms). First, we used a stratified random sampling to select fattening pig farms from the whole population of fattening pig farms. One thousand farms with fattening pigs were selected from the four different groups of fattening pig farms cited in the previous paragraph (targeting ~100 farms, based on no more than a 10% positive response to participate in the study, which represented the maximum number of farms for which we could realistically conduct a farm visit in the time available for the project). To avoid the overrepresentation of the smallest farms, we used a stratified random sampling in which the percentage of farms selected in each stratum was equivalent to the corresponding percentage of pigs in each group (1–4) for the whole population. This strategy was used to select the larger herds and reduce the number of farms from group 1 and group 4, which were of peripheral interest to the study. The CPH number of the selected farms was communicated to the AHDB, the custodian of farmer identity, which sent a letter to the selected pig producers to invite them to participate in the study. The farmer name and farm location remained confidential to the mailing body. We sent several reminders to the farmers by regular mail and email through AHDB but, due to the low percentage of replies to the initial mailing, we had to recruit additional fattening farms by advertising online on the National Pig Association (NPA) website and contacting farms that had previously participated in similar studies. The breeding farms were not originally part of the objective of this study. However, considering the number of breeder-fatteners visited in the fattening pig farm sample, a breeding farm sample was constituted afterward, which included the breeding-fattening farms from the fattening pig farm sample and was completed by additional specialized breeding farms that agreed to participate to our study. These farms were not randomly selected, having participated in previous studies for Newcastle University and agreeing to be contacted to participate in further studies.

### Data Collection

**Figure 1:**
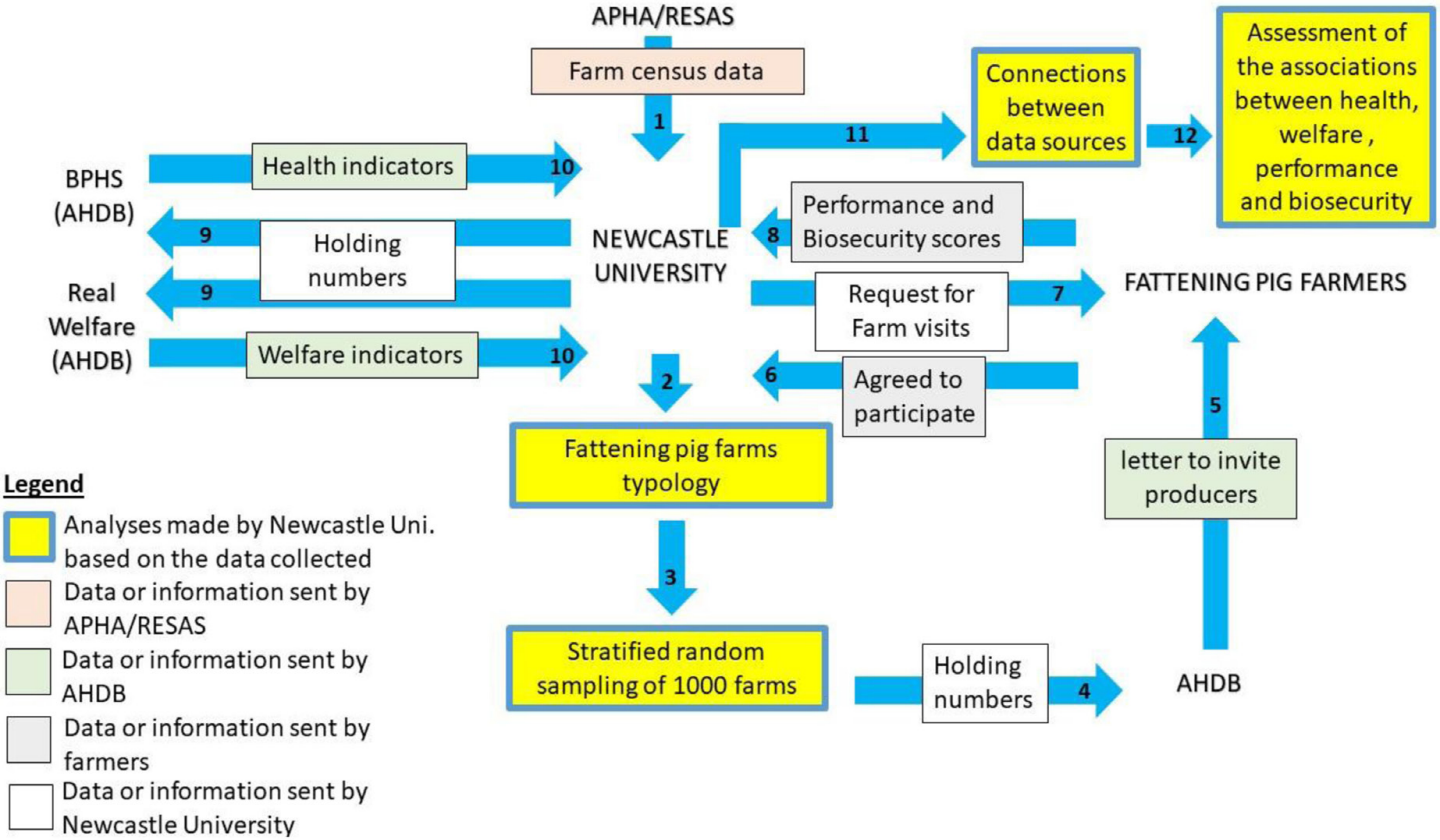
Diagram representing the sampling and data collection. Data about biosecurity, health, welfare, and performance were collected on-farm and from the Agricultural and Horticultural Development Board (AHDB) databases [British Pig Health Scheme (BPHS) and Real Welfare schemes]. The sampling was based on farm census data from the Animal and Plant Health Agency (APHA) and the Rural and Environment Science and Analytical Services (RESAS). The numbers on the graph indicate the order of the different steps in the data collection and the data analysis.

If the farmer agreed to participate in the study by a written agreement that they sent to Newcastle University by post or email, the first step was to complete a biosecurity questionnaire (online or paper version) and communicate their name, address and phone number. We then arranged a convenient time for a farm visit to confirm the accuracy of the responses to the biosecurity questionnaire and to collect performance data (Table 1) for the year before the visit. Herd visits took place between July 2015 and December 2016. After the visits, the prevalence of welfare indicators for the sample of fattening pig farms, collected during quarterly veterinary visits in 2015 and 2016, were acquired from the database of the AHDB Pork “Real Welfare” scheme (18) and the prevalence of different lesions recorded at the abattoir were acquired, for all batches assessed in 2015 and 2016, from the database of the AHDB Pork “BPHS”^1^ (Table 1). The connection between the farm ID and the BPHS and Real Welfare databases was processed by AHDB to maintain confidentiality. A diagram which summarizes the sampling and the data collection for fattening pig farms is presented in Figure 1.

**Table 1:** Production data for the study farms, collected during a farm visit, and health and welfare indicator data collected from the British Pig Health Scheme and Real Welfare databases of Agricultural and Horticultural Development Board Pork.

*Performance data for breeding pigs*	
PB	Piglets born^a^
PBA	Piglets born alive^a^
PW	Piglets weaned^a^

*Performance data for fattening pigs*	
ADG	Average daily weight gain^b^
FCR	Feed conversion ration^b^
MOR	Post weaning mortality^b^

*Real Welfare data*	
hosp	Pigs requiring hospitalization^c^
lam	Lameness^c^
stl	Severe tail lesions^c^
sbm	Severe body marks^c^

*British Pig Health Scheme data*	
ep	Enzootic pneumonia^d^
pl	Pleurisy^d^
pc	Pericarditis^d^
pt	Peritonitis^d^
ms	Milk spot^d^
hs	Hepatic scarring^d^
pd	Papular dermatitis^d^
tail	Tail-bitten^d^
viral	Viral-type distribution^d^
ppa	Pleuropneumonia—acute^d^
ppc	Pleuropneumonia—chronic^d^
abscess	Abscess^d^
pyaemia	Pyaemia^d^
ep score	Score enzootic pneumonia^e^
pl score	Score pleurisy^e^
pd score	Score papular dermatitis^e^

*^a^Average number per litter for the farm*.

*^b^Average for the farm from weaning to slaughter*.

*^c^Estimated mean farm prevalence for 2015 and 2016 based on repeat samples of pigs selected to be representative of the farm*.

*^d^Estimated mean farm prevalence for 2015 and 2016 based on repeat samples of pigs selected at the abattoir*.

*^e^Estimated mean scores for 2015 and 2016 based on repeat samples of pigs selected at the abattoir. For ep score, each lobe is designated a score, giving a total score between 0 and 55 according to severity, pl score is scored (0–2) and pd score is scored (0–3) also according to severity*.

### Biosecurity Scoring Tool

The level of farm biosecurity was assessed using a risk-based scoring tool which was a slightly modified version of “Biocheck-UGent™”^2^ (19). The risk-based scoring tool is a questionnaire with 130 questions. Fifteen questions are used to collect contact information and data about herd characteristics [herd ID, presence of other animals, number of breeding pigs, number of weaners, number of fattening pigs, number of boars, years of experience, and people working in full time equivalents (FTE)]. The answers to all of the other 115 questions were translated into a score between 0 and 10 according to the relative importance of the question regarding farm biosecurity and disease prevention (19). The 115 questions were grouped into 12 different subcategories: A. Purchase of animals and semen; B. Transport of animals, removal of manure/dead animals; C. Feed, water and equipment supply; D. Personnel and visitors; E. Vermin/bird control; F. Environment and region; G. Disease management; H. Farrowing period; I. Nursery, J. Fattening pigs; K. Measures between compartments and the use of equipment; and L. Cleaning and disinfection. The subcategories have specific weight factors according to the relative importance for disease prevention, giving a score for external biosecurity (EXT) based on the score of the categories A–F and a score for internal biosecurity (INT) based on the score of the categories G–L. The total biosecurity (TOT) score was the average of the internal and external biosecurity score. All questions for each category can be found by accessing the Biocheck-UGent scoring tool online.

### Statistical Analysis

#### Farm Description

Using the methodology of classification described in Section “Sampling,” the sample of farms that participated in the study was compared with the proportion of farms in the different groups in the national population using Fisher or chi-square tests. The null hypothesis H0 was “No difference in the proportion of farms in the different groups between the whole population and the sample.” If *P* < 0.05 the null hypothesis was rejected.

For the sample of farms (fattening farms and breeding farms), the correlations between herd characteristics were identified. First, a Shapiro test was used to assess the normality of the different variables. When *P* > 0.05 in the Shapiro test, Pearson correlation coefficients were calculated. When *P* < 0.05 in the Shapiro test for at least one of the variables, Spearman rank correlation coefficients were calculated. The correlation was considered significant if the correlation coefficient *r* > |0.3| and *P* < 0.05, and considered strongly correlated if *r* > |0.6| and *P* < 0.05.

#### Biosecurity Score and Farm Types

We assessed the association between internal, external and total biosecurity scores (dependent variables) with the different independent variables related to farm characteristics [farm system, presence of other animals, number of breeding pigs, number of weaners, number of fattening pigs, number of boars, years of experience, and number of people working full time (as FTE)] using univariate linear regression. All the variables with *P* < 0.25 were retained for a multivariate regression. We used a stepwise variable selection to build the final model, and we also tested the interactions between the dependent variables. The association between the dependent variables and the independent variables was considered significant if *P* < 0.05. The model was also assessed for multicollinearity for the multivariate analysis. Finally, we calculated the biosecurity score for each production type (breeders, weaners, and fatteners; breeders only or breeders and weaners; weaners and fatteners; and fatteners only).

#### Interconnections between Biosecurity Scores, Health Indicators, Welfare Outcomes, and Production Performance

The correlations between total and individual scores of internal and external biosecurity were assessed separately for the fattening pig farms and the breeding pig farms. The correlations between total, internal and external biosecurity score, the health indicator prevalence from BPHS data, the welfare outcomes and the production performance (Table 1) were assessed using Pearson or Spearman correlations.

To provide an overview of the connection between biosecurity score, the BPHS data, welfare outcomes, and production performance in the sample of fattening pig farms, a principal component analysis (PCA) was used. For the PCA, 13 variables were considered: the main biosecurity scores (INT, EXT, and TOT), production performance [ADG, feed conversion ratio (FCR), and MOR], welfare outcomes from Real Welfare dataset [hosp, lam, severe tail lesions (stl), and severe body marks (sbm)], the more prevalent abattoir lesions from BPHS dataset [enzootic pneumonia (ep) and pl], and the prevalence of tail-biting lesions (tail) (Table 1). Tail-biting lesions were included to allow assessment of the connection with the on-farm prevalence of the welfare outcome of stl in the Real Welfare dataset. We used normalized variables as all these variables have different units.

We imputed the 12 missing entries for the AHDB data using the iterative PCA algorithm. First, the number of dimensions was estimated and we imputed the missing values using the number of dimensions previously calculated. Based on this calculation, we estimate the eigenvalue for each component. Finally, a bootstrapping method was used to assess the variability with which missing values can be predicted and assess the stability of the PCA. The two first components from the PCA, considered as the most discriminating, were selected and the cumulative percentage of inertia was calculated for these components. Then we plotted the farms and the variables on the factor map. We used an Ascendant Hierarchical Clustering, based on the selected principal components of the PCA, to place individual farms into different clusters. The clustering was achieved based on the “Ward” criteria. Then, the sum of the within-cluster inertia was calculated for each partition. The number of clusters corresponds to the partition with the higher relative loss of inertia [*i*(clusters *n* + 1)/*i*(cluster *n*)] which was identified according to the length of the tree branches on a hierarchical tree. ANOVA or Kruskal–Wallis tests and *post hoc* pairwise comparisons using Tukey and Kramer (Nemenyi) tests with a Tukey-Dist approximation were used to assess the differences between clusters in production performance, biosecurity scores, the prevalence of the BPHS lesions (including those not used in the PCA), and of the different welfare outcomes. Differences were considered significant if *P* ≤ 0.05.

Data processing was carried out using Microsoft Access Office Professional Plus 2010 and Microsoft Excel Office Professional Plus 2010 to create the datasets. The data were analyzed with RStudio for R-3.1.0 software for Windows (64 bit).

## Results

### Sample of Fattening and Breeding Pig Farms

The number of farms in each classification group for the whole population and in the study sample is reported in Tables 2 and 3. As expected, the proportion of fattening pig farms in the four different groups was different between the whole population figure and the sample (*P* < 0.05), since we sampled according to the proportion of pigs rather than farms. Thus the sampled fattening pig farms belonged mainly to group 2 (0 breeding pigs and ≥400 fattening pigs) and group 3 (≥100 breeding pigs and ≥400 fattening pigs); the sample represents mainly the fattening pig farms with the bigger herds. The proportion of breeding pig farms in the two different groups was also different between the whole population figure and the sample (*P* < 0.05). The sample had a higher percentage of farms from group B (breeder-fatteners), which represented the larger breeding herds in the pig farm population. In such a small sample, farms from group 1 and 4 could have been excluded as they did not impact the analysis. However, to have a sample representative of the pig population, we considered it important to keep these groups of farms.

**Table 2:** Number of farms and number of pigs per classification group^a^ in the whole population (Pop.) and in the study sample (Samp.) of fattening pig farms.

	Group 1 Pop.	Group 1 Samp.	Group 2 Pop.	Group 2 Samp.	Group 3 Pop.	Group 3 Samp.	Group 4 Pop.	Group 4 Samp.	Total Pop.	Total Samp.
Number of fattening pig farms	1,848	1	806	19	603	17	10,556	3	13,813	40
Percentage of fattening pig farms	13.4	2.5	5.8	47.5	4.4	42.5	76.4	7.5	100	100
Total number of pigs	5,691	4	1,158,028	62,976	1,066,601	92,447	295,661	2460	2,525,961	157,887
Percentage of pigs	0.2	<0.01	45.8	39.9	42.2	58.6	11.4	1.5	100	100

*The whole population figure is based on the data collected in 2010 for England, Scotland, and Wales. The sample is based on the farms visited in Great Britain between 2015 and 2016*.

*^a^Group 1: small fatteners (no breeding pigs and less than 10 other pigs); group 2: large fatteners (no breeding pigs, at least 400 other pigs); group 3: large breeder-fatteners (at least 400 other pigs and 100 breeding pigs); and group 4: other farms that cannot be classified in the 3 other groups because the number of breeders and fatteners present in the farm do not match with their definition*.

**Table 3:** Number of farms and number of pigs per classification group^a^ in the whole population (Pop.) and in the study sample (Samp.) of breeding pig farms.

	Group A Pop.	Group A Samp.	Group B Pop.	Group B Samp.	Total Pop.	Total Samp.
Number of breeding pig farms	2,698	6	3,512	22	6,210	28
Percentage of breeding pig farms	43.4	21.4	56.6	78.6	100	100
Number of breeding pigs	106,668	5,658	367,782	8,755	474,450	14,413
Percentage of breeding pigs	22.5	39.3	77.5	60.7	100	100

*The whole population figure is based on the data collected in 2010 for England, Scotland, and Wales. The sample is based on the farms visited in Great Britain between 2015 and 2016*.

*^a^Group A: breeding only; group B: breeding-fattening farms*.

### Description of the Sample of Farms and Connection of the Data Sources

We recruited 46 farms for the study, providing one sample of 40 fattening pig farms and one sample of 28 breeding farms, with 22 farms (breeder–fatteners) that were included in both categories. From the 1,000 farms initially sampled, only 902 were present in the AHDB dataset. Only 35 farms recruited by the stratified random sampling accepted to participate in our study; this was lower than the expected participation. Five additional fattening farms were recruited by advertising online on the NPA website or were contacted because they were involved in a previous study. Twenty-two breeding farms were recruited from the fattening pigs sample and six breeding farms were additionally recruited through advertising or directly contacted. For the 22 fattening-breeding farms, the part of the questionnaire dedicated to the breeding pigs was completed during the visit concomitantly to the information collected about fattening pigs. An additional six visits were organized for the additional six breeding farms. Of the 40 fattening farms in the final study sample, only 28 could be identified by AHDB in the Real Welfare and BPHS databases.

Among the 46 farms (fattening pig farms and breeding pig farms), 16 farms had other animals: 14 had sheep or lambs, 10 had beef or cattle, and 1 had poultry. The description of herd characteristics (first part of the questionnaire) is reported in Table 4. The average number of fattening pigs was higher than the average number of fattening pigs for the whole population of fattening pig farms with more than 10 pigs in England [513 fattening pigs in 2016 (20)]. This suggests than our sample is more representative of the farms with larger herd size. None of the variables related to herd characteristics were normally distributed. As would be expected, there were strong intercorrelations between the number of boars, the number of breeding pigs, weaner pigs, and number of employees (*r* > 0.6, *P* < 0.05), but the number of employees was not correlated to the number of fattening pigs or to the number of years of experience of the farmer (*r* < 0.3, *P* > 0.05).

**Table 4:** Description of the herd characteristics for the study sample of fattening and breeding pig farms in Great Britain.

	Mean	SD	Median	Min	Max
**Fattening pig farms^a^**					
Number of breeding pigs	219	269	105	0	1,000
Number of weaners	1,166	1,194	904	0	4,600
Number of fattening pigs	2,003	1,397	1,700	2	6,200
Number of boars	3	4	3	0	15
Years of experience	30	13	30	2	60
Number of employees [full time equivalents (FTE)]	2.8	1.7	2	0.6	7
**Breeding pig farms^b^**					
Number of breeding pigs	515	370	435	85	1,700
Number of weaners	1,776	1,443	1,500	0	5,400
Number of fattening pigs	1,553	1,567	1,425	0	6,200
Number of boars	8	7	6	3	33
Years of experience	31	12	30	3	60
Number of employees (FTE)	4.0	1.6	4.0	1.5	7

*^a^40 fattening pig farms (specialized fatteners and breeder-fatteners)*.

*^b^28 breeding pig farms (specialized breeders and breeder-fatteners)*.

### Interrelationships between Biosecurity Scores, Health Indicators, Welfare Outcomes, and Production Performance

#### Description of Biosecurity Scores, Health Indicators, Welfare Outcomes, and Production Performance

The different biosecurity scores for all pig farms (breeding farms and fattening pig farms) are presented in Table 5. The comparison between fattening farm biosecurity scores and breeding farm biosecurity scores should be made with caution as breeding-fattening farms are included in both categories. The total biosecurity score ranged from 40.1 to 89.5 for the fattening farms and from 43.8 to 83.8 for the breeding farms (on the scale of 0–100). The highest mean subcategory score was for score A (purchase of animals and semen) and the lowest mean score was for score H (farrowing period).

**Table 5:** Description of Internal, External biosecurity score, their respective subcategory scores, and the total biosecurity scores for a sample of fattening and breeding pig farms in Great Britain visited in 2015–2016.

	Fattening pig farms (*n* = 40)^c^					Breeding pig farms (*n* = 28)^d^				
	Mean	SD	Median	Min	Max	Mean	SD	Median	Min	Max
A. Purchase of animals and semen^a^	92.1	9.31	95.7	72.8	99.8	90.8	10.6	96.7	73	99.8
B. Transport of animals, removal of manure/dead animals^a^	76.4	11.3	78.3	41.6	95.7	77.3	10.6	78.7	54	95.7
C. Feed, water and equipment supply^a^	55.9	21.8	53.6	14.3	100	55.0	23.4	51.8	14	100
D. Personnel and visitors^a^	63.5	19.9	64.7	14.7	100	66.3	20.8	67.6	18	100
E. Vermin/bird control^a^	67.3	21.5	72.8	27.3	100	61.4	21.8	63.7	27	100
F. Environment and region^a^	85.9	19.3	85	10	100	88.2	15.2	90.0	30	100
External biosecurity score	74.5	7.89	74.8	54.5	90.5	74.4	6.95	74.8	55	84.5
G. Disease management^b^	80.3	20.7	80	0	100	80.0	21.8	80.0	0	100
H. Farrowing period^b^	27.9	26.4	33.9	0	78.5	43.1	18.4	39.3	0	67.8
I. Nursery^b^	43.2	32	53.6	0	89.3	57.2	23.8	60.7	0	89.3
J. Fattening pigs^b^	56.7	36.3	78.5	0	100	47.4	36.6	42.8	0	100
K. Measures between compartments and the use of equipment^b^	49.3	18.3	46.4	14.3	100	45.6	15.4	46.4	17.9	85.7
L. Cleaning and disinfection^b^	66.8	24.5	72.5	0	100	59.2	24.1	61.3	0	95.0
Internal biosecurity score	60.5	14.4	59.6	25.7	89.9	55.9	12.0	57.1	29.0	87.0
Total biosecurity score	67.5	10	68.3	40.1	89.5	65.1	8.15	65.4	43.8	83.8

*^a^External biosecurity subcategories*.

*^b^Internal biosecurity subcategories*.

*^c^All farms having fattening pigs in our initial sample*.

*^d^All farms having breeding pigs in our initial sample*.

For the performance variables, only FCR and mortality were not normally distributed. For the fattening pig farms, the mean ADG, FCR, and MOR were 772 (±104) g/day, 2.45 (±0.39) kg of feed/kg of weight gain (median = 2.36, minimum value = 2, maximum value = 3.82), and 3.6 (±1.5)% (median = 3.02, minimum value = 1.6, maximum value = 6.8), respectively. For the breeding pig farms, the mean piglets born (PB), piglets born alive (PBA), and piglets weaned (PW) per litter were 13.67 (±0.88), 12.89 (±0.73), and 11.47 (±0.74) respectively. The description of the mean prevalence of the welfare outcomes for 2015–2016 is reported in Table 6.

**Table 6:** Number of pigs assessed and prevalence (%) of pigs requiring hospitalization, lame pigs, pigs with severe tail lesions (stl), and severe body marks (sbm) for 2015–2016 in the study sample of fattening pig farms (*n* = 28).

	Mean	SD	Median	Min	Max
Number of pig assessed	3,028	2,208	2,840	300	8,858
Pigs requiring hospitalization (%)	0.03	0.04	0	0	0.14
Lameness (%)	0.1	0.23	0	0	0.91
stl (%)	0.23	0.43	0	0	1.51
sbm (%)	0.23	0.31	0.11	0	1.04

The mean prevalence of the different lesions recorded in BPHS data during the 2 years of the farm visits (2015 and 2016) and the mean lesion scores for ep, pleurisy, and papular dermatitis are reported in Table 7. The two most common lesions were ep and pleurisy (pl), recorded in 15.30 and 4.72%, respectively, of pigs assessed.

**Table 7:** Prevalence (%) of the 13 pathologies recorded in British Pig Health Scheme data and mean scores of enzootic pneumonia (ep), pleurisy, and papular dermatitis for a sample of fattening pig farms in Great Britain visited in 2015–2016 (*n* = 28).

	Mean	SD	Median	Min	Max
EP-like lesions (%)	15.30	11.65	12.61	0	52.17
Pleurisy (%)	4.72	5.75	3.00	0	28.78
Pericarditis (%)	1.79	1.12	1.55	0	4.65
Peritonitis (%)	0.15	0.28	0.01	0	1.10
Milk spots (%)	0.05	0.12	0.00	0	0.45
Hepatic scarring (%)	1.40	2.46	0.38	0	9.18
Papular dermatitis (%)	1.30	4.07	0.00	0	17.35
Tail-bitten (%)	0.67	1.99	0.00	0	8.19
Viral-type distribution (%)	0.17	0.35	0.00	0	1.30
Pleuropneumonia—acute (%)	0.12	0.19	0.00	0	0.65
Pleuropneumonia—chronic (%)	0.08	0.21	0.00	0	1.08
Abscess (%)	0.16	0.25	0.02	0	1.17
Pyaemia (%)	0.08	0.15	0.00	0	0.50
Score ep^a^	3.11	2.86	2.69	0	14.17
Score pleurisy^a^	0.11	0.10	0.09	0	0.45
Score papular dermatitis^a^	0.04	0.12	0.00	0	0.58

*^a^ep score is scored (0–55), pl score is scored (0–2), and pd score is scored (0–3) according to the severity of the lesions*.

#### Associations between Biosecurity Scores and Farm Types

The only scores which were normally distributed (Shapiro test *P* > 0.05) were internal biosecurity score, external biosecurity score, total biosecurity score, score C (feed, water, and equipment supply), and score D (personnel and visitor). The correlations between different biosecurity scores for the fattening farms are reported in Table S1 in Supplementary Material, and those for the breeding farms in Table S2 in Supplementary Material. External biosecurity score was strongly correlated to scores for the subcategories: B. Transport of animals, removal of manure/dead animals; C. Feed, water, and equipment supply; D. Personnel and visitor (*P* < 0.05, *r* > 0.6). Total biosecurity and internal biosecurity scores were strongly correlated and were also strongly correlated to external biosecurity score and scores for the subcategories: J. Fattening pigs; K. Measures between compartments and the use of equipment; and L. Cleaning and disinfection (*P* < 0.05, *r* > 0.6).

After backward selection, only the variable “other animals in the farm” was included in the final model with total biosecurity as dependant variable. The total biosecurity score was 6.2/100 units lower when other animals where present in the farm (*P* < 0.05). Based on the multivariable analysis, only borderline results were found for internal biosecurity. The internal biosecurity score tended to be 8.1 U lower when other animals were present in the herd (*P* = 0.056) and increased by 0.3 when the fattening pig herd size increased by 100 (*P* = 0.054). No significant association was identified between the external biosecurity scores and farm characteristics (*P* > 0.05). The results showed that the variable “farm type” did not have a significant association with any biosecurity scores, although the univariate analysis showed a borderline result with higher internal biosecurity score for the farms with fatteners only (*P* = 0.06). The mean biosecurity scores for each farm type, as specified in this analysis, are reported in Table 8.

**Table 8:** Mean and SD of internal (INT), external (EXT), and total (TOT) biosecurity scores for the different types of farm.

	Breeders, weaners, and fatteners (*N* = 22)	Breeders only or breeders and weaners (*N* = 6)	Weaners and fatteners (*N* = 9)	Fatteners only (*N* = 9)
EXT	73.0 (±7.17)	79.3 (±2.86)	74.1 (±12.22)	75.4 (±6.01)
INT	55.3 (13.3)	58.1 (5.42)	66.0 (20.63)	69.3 (7.96)
TOT	64.2 (8.87)	68.7 (2.97)	70.0 (16.11)	72.3 (5.13)

*The scores are out of a maximum of 100 for a sample of pig farms in Great Britain visited in 2015–2016*.

#### Interconnections between Biosecurity Scores, Health Indicators, Welfare Outcomes, and Production Performance for Fattening and Breeding Pig Farms

The correlations between production performance, biosecurity scores recorded during the farm visits, and the mean prevalence for health indicators and welfare outcomes for 2015–2016 are reported in Table S3 in Supplementary Material for the fattening herds and in Table S4 in Supplementary Material for the breeding herds. In fattening herds, the percentage of mortality was strongly correlated to the percentage of lameness (*r* = 0.67, *P* < 0.001), the percentage of ep was strongly correlated to the percentage of pl (*r* = 0.66, *P* < 0.001), the ep score was strongly correlated to the percentage of ep (*r* = 0.79, *P* < 0.001), the pl score was strongly correlated to the percentage of pl (*r* = 0.9, *P* < 0.001), the percentage of peritonitis was strongly correlated to the percentage of papular dermatitis (*r* = 0.64, *P* < 0.001), the percentage of hepatic scaring was strongly correlated to the percentage of tail-bitten pigs (*r* = 0.62, *P* < 0.001), and the percentage of abscess was strongly correlated to the percentage of pyaemia (*r* = 0.62, *P* < 0.001). In breeding herds, the number of PB, the number of piglet born alive, and the number of piglet weaned were strongly intercorrelated (*r* > 0.6, *P* < 0.001). All correlation coefficients are reported in Tables S3 and S4 and Figures S1 and S2 in Supplementary Material.

**Figure 2:**
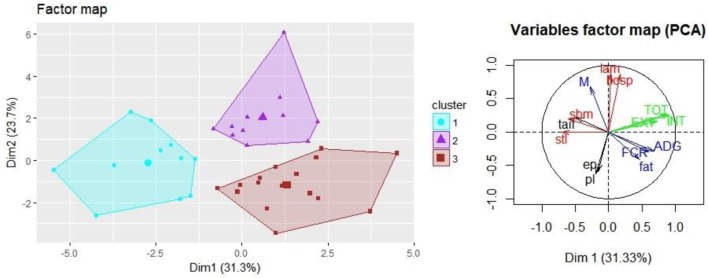
Principal component analysis (PCA) plot of the fattening farms (individual farms) and the variables on the two first components (CP1: 31.33%; CP2: 23.66%). Biosecurity [external (EXT), internal (INT), and total (TOT) biosecurity] is represented in green. The number of fattening pigs (fat) and the performance [average daily weight gain (ADG), feed conversion ratio (FCR), and mortality (MOR)] are represented in blue. Welfare outcomes [% pigs requiring hospitalization (hosp), lame pigs (lam), pigs with severe tail lesions (stl), and severe body marks (sbm)] are represented in red. Health indicators [% of enzootic pneumonia-like lesions (ep), pleurisy (pl), and tail-bitten lesions (tail)] are represented in black. A hierarchical clustering on the result of the PCA confirmed the partition in three clusters as the partition with the higher relative loss of inertia.

A PCA was used to assess the interconnections between biosecurity scores, health indicators, welfare outcomes, and production performance for 40 fattening pig farms. The plot of the PCA on the two first components for the farms and the variables is presented in Figure 2. The first component explained 31.33% of the total variance and the second component 23.66% of the total variance, giving a cumulative percentage of inertia for the two first components of 54.99%. The first axis was mainly represented by the biosecurity scores, ADG, severe tails lesions, and sbm and was the most discriminative. This highlighted a separation between farms with high biosecurity and higher ADG and those with higher prevalence of stl and body marks. The biosecurity scores, the number of fattening pigs, and production performance were grouped together on the right side of the PCA plot while the percentages of lameness, pigs requiring hospitalization and mortality were grouped on the upper side, the percentage of sbm and tail lesions was grouped on the left side and the percentage of ep and pleurisy on the lower side. A partition in three clusters was inferred from the length of the branches of the dendogram and can be visualized on Figure 2. The bootstrapping method used to assess the PCA stability plotted the variables on the two first factorial axes in slightly different positions but relatively close to each other, suggesting sufficient stability of the PCA to interpret the output.

Cluster 1 had lower external, internal, and total biosecurity scores compared with clusters 2 and 3 (*P* ≤ 0.05). Cluster 1 had higher prevalence of peritonitis than cluster 2. Cluster 1 had lower (better) FCR, a smaller number of fattening pigs in the unit and higher prevalence of stl and sbm compared with cluster 3 (*P* ≤ 0.05). Cluster 2 had higher mortality and prevalence of lameness than cluster 3 (*P* ≤ 0.05). Cluster 3 had higher ADG than cluster 1 and cluster 2 (*P* ≤ 0.05). The variables hosp, ep, pl, tail, pc, ms, hs, pd, viral, ppa, ppc, abscess, and pyaemia were not significantly different between the three clusters (*P* > 0.05) (Table 9).

**Table 9:** Mean and SD of biosecurity scores, health indicators, welfare outcomes, and production performance, and of the study sample of fattening pig farms in Great Britain according to three clusters derived from the PCA analysis (based on the active variables).

	Cluster 3 (*n* = 18)		Cluster 2 (*n* = 11)		Cluster 1 (*n* = 11)	
	Mean	SD	Mean	SD	Mean	SD
**Active variables**						
Fattening pigs	2,733^b^	1,513	1,578^a,b^	1,220	1,232^a^	646
Average daily weight gain	834^b^	60	734^a^	54	718^a^	46
Feed conversion ration	2.55^b^	0.46	2.44^a,b^	0.19	2.15^a^	0.17
Mortality	2.82^b^	0.71	5.22^a^	1.18	3.96^a,b^	1.64
Pig requiring hospitalization	0.01	0.03	0.12	0.02	0.03	0.04
Lameness	0.02^b^	0.05	0.62^a^	0.42	0.05^a,b^	0.06
Severe tail lesions	0.03^b^	0.06	0.29^a,b^	0.18	0.63^a^	0.64
Severe body marks	0.08^b^	0.13	0.09^a,b^	0.02	0.58^a^	0.35
External biosecurity score	77^b^	8	76^b^	5	68^a^	8
Internal biosecurity score	65^b^	13	70^b^	10	46^a^	12
Total biosecurity score	71^b^	9	73^b^	6	57^a^	9
Enzootic pneumonia	16.49	13.11	9.43	4.41	21.08	12.62
Pleurisy	5.33	7.17	1.95	1.30	6.45	5.64
Tail-bitten pigs	0.26	0.86	0.07	0.14	2.28	3.69

**Supplementary variables**						
Pericarditis	1.85	1.22	1.60	1.04	2.14	0.89
Peritonitis	0.12^a,b^	0.15	0.01^b^	0.04	0.42^a^	0.46
Milk spots	0.06	0.12	0.01	0.04	0.07	0.17
Hepatic scarring	0.86	1.15	0.33	0.60	3.01	4.11
Papular dermatitis	0.97	3.50	0.00	0.00	3.65	6.67
Viral-type distribution	0.25	0.43	0.12	0.33	0.09	0.17
Pleuropneumonia—acute	0.14	0.19	0.00	0.00	0.22	0.26
Pleuropneumonia—chronic	0.15	0.30	0.01	0.02	0.03	0.05
Abscess	0.25	0.33	0.04	0.10	0.14	0.13
Pyaemia	0.12	0.19	0.02	0.04	0.09	0.12

*^a,b^Means in the same row with different letters are significantly different (*P* < 0.05)*.

## Discussion

Our study aimed at identifying possible connections between on-farm data and large-scale industry databases holding information about health and welfare for commercial pig farms in Great Britain. During farm visits, we collected data about pig performance and assessed the level of biosecurity in fattening and breeding farms. Subsequently, we identified interconnections between these data and the mean prevalence of welfare outcomes from the Real Welfare Scheme and health indicators from the BPHS for fattening pigs. This analysis enables us to propose a hypothesis regarding the interconnection between health, welfare, performance, and biosecurity in commercial pigs farms that would need to be verified through additional studies to extrapolate the results of this analysis to a wider population of pig farms in the UK or pig farms in other countries.

### The Challenge of Secondary Data Analysis

#### Limitations of Secondary Data Analysis

Secondary data analyses were used in this study because direct implementation for such a study would have been logistically and financially impossible in 2 years (3, 4). However, several difficulties arise from secondary data analysis. Secondary data are collected neither for the purpose of research nor regarding a specific study design and, as was the case in this study, might require the combination of several data sources. The presence of missing values or necessary imputations might impair the data quality and the ability to conduct analysis with the data available. Data about pig health and welfare are regularly recorded by the Real Welfare and BPHS for the purpose of informing farm management decisions and aiding farm improvement, and cover a large majority of the fattening pig farms in Great Britain (6, 18, 21). There is a risk of discrepancies in the data recorded as nationwide data are recorded by several observers. However, the standardized procedure and the training provided in each scheme were designed to minimize observer bias and were assessed in previous studies (18, 21). Our study illustrates the challenge of using secondary data related to the pig industry and highlights the challenges regarding the data collection, the connection of different data sources and the design of a random sampling from the whole population of pig farms which is large enough to be representative of the commercial pig farms in the UK. It illustrates the difficulty to implement a detailed and time consuming survey in a population of farmers previously unknown and to collect additional information from existing databases for these farms. Connecting the data collected to the BPHS and Real Welfare database resulted in selection bias, as only 28 of the 40 fattening pig farms who participated could be found in both databases. This highlights the considerable room for improvement in organization of industry data needed to conduct secondary data analysis about pig farms based on several data sources.

#### Sample Size

The target of most studies is to analyze a sample representative of the population (22). Targeting to select the intensive pig farms with the larger herds, we decided to use a stratified random sampling proportionate to the number of fattening pig produced in each stratum. However, in our final study, selection biases can be identified. Despite the possibility to select a stratified random sampling from the full database of pig farms in Great Britain, we were not able to access the farm identification and address for confidentiality reasons. As a consequence, a first selection bias occurred due to the exclusion of all farms not registered in the AHDB database, which was a requirement to be able to invite farmers to participate in our study. Another selection bias was due to a very high percentage of pig farmers who did not reply to the invitation or declined to participate. This level of non-response reduced the level of precision and increased the risk of non-representativeness (23). Higher response rates have been achieved in other agricultural studies involving pig farms (24, 25), but their farmers were not pre-selected from the whole national population. Moreover, the length of the biosecurity questionnaire, followed by a mandatory farm visit, might have dissuaded some farmers from participating.

We succeeded to recruit farms of interest (large breeders-fatteners or specialized fattening pig farms with larger herds which produce most of the fattening pigs in Great Britain), but the final sample size was limited. Considered the low prevalence of welfare outcomes and the non-negligible SD of most of the variables, the results should therefore be extrapolated only with great care to a wider population of pig farms. However, our analysis remains accurate for the sample of farms that has been considered and the results remain valid as material to elaborate hypotheses. This could represent the first step of a grounded theory study which then needs to be completed by further studies in the UK or different countries to challenge these primary results. The outcome highlights the need in studies of this nature to find an optimal balance between the quantity of information per farm and the sample size. Considering the value of the output that could be produced, improving the possible connection between different data sources would be of great benefit for the pig industry in the UK. This would enable future work to repeat this study with a larger number of farms.

### Biosecurity in Fattening and Breeding Herds

#### Assessment of Biosecurity in Commercial Pig Farms in the UK

Biocheck.UGent™ was used for the first time in the UK. Biosecurity comprises a set of measures targeting the protection of pig herds from the introduction and spread of infectious diseases (26–29). This tool has previously been used in different farm studies and several other countries (19, 30–32), and its reliability to quantify and compare biosecurity between pig herds has been demonstrated by Laanen et al. (30). Our study suggests room for improvement in the level of biosecurity for pig farms in Great Britain. The mean internal biosecurity score was lower than the mean external biosecurity score, as in previous studies (19, 31, 33), and the scores for many subcategories were lower than in these studies. A higher external biosecurity score can be explained because the farmers were generally aware about the risk of contamination and the threat of diseases from outside the farms, especially for the diseases regulated by control programs (34, 35). By contrast, the lower internal biosecurity score indicates that the risk of contamination inside the farm, arising through daily management practices, seemed to be underestimated. Garthford et al. (36) showed that there is little concern about risk from unseen diseases. Vets should use their authoritative position to promote better internal biosecurity and good awareness of disease risks by transferring knowledge about biosecurity (25, 36), and special attention should be given when other animals are present in the farm as this was associated with lower biosecurity scores.

In the univariate analyses, internal biosecurity scores were higher for larger herds of fattening pigs and specialized fattening farms, while total biosecurity score was strongly correlated to measures between compartments and the use of equipment, and cleaning and disinfection, suggesting a good all-in/all-out (AIAO) system for the farms that obtained a higher total biosecurity score. Generally, specialized fattening pig herds are more likely to have larger pig herds and to adopt an AIAO system, which contributes to good biosecurity (17, 29, 37). However, internal biosecurity was not significantly different for specialized fattening farms in the multivariate analysis; pointing to the influence of other factors. This suggests room for improvement of the level of biosecurity which does not depend only on farm type.

Breeding farms had lower internal biosecurity compared with fattening pig farms. The total biosecurity score of breeding farms was strongly correlated to the internal biosecurity score and the cleaning and disinfection scores, suggesting that hygienic measures were the cornerstone of the breeding farms achieving a high level of biosecurity. Measures between compartments and the use of equipment, which largely refer to piglet manipulation, mixing of piglets from different sources, proper use of overalls, cleaning of boots, hands and materials (19), had one of the lowest scores, highlighting areas where farms could seek biosecurity improvement (31).

The type of buildings may impair the implementation of internal biosecurity measures, such as AIAO or an increase of space allowance, and the perceived cost of the biosecurity measures might also influence the likelihood of adopting these measures (37). Several studies have shown the reluctance to adopt certain measures considered to be difficult to implement or with lack of trust in their effectiveness or relevance (38, 39), but the increase of awareness regarding specific biosecurity measures should encourage the popularization of all biosecurity measures and beneficial changes in management. Despite possible structural limitations, Laanen et al. (19) suggest that the improvement of internal biosecurity constitutes a good starting point, which was confirmed by this study. Providing their biosecurity score to farmers could enable them to compare the score of their farm with the national average, identify easily their weaknesses and encourage them to make changes to perform better in the future.

#### Interconnection between Biosecurity and Health and Performance

Through this analysis, several interconnections between the level of biosecurity and indicators of health, welfare or performance were identified. The farms from cluster 1, with lower ADG and higher prevalence for some welfare and health indicators compared with the other clusters, also had lower biosecurity scores. Biosecurity appears of great importance to maintain good production results, health and welfare and, by extension, to protect the economy, environment, and public health (27, 40). A recent study showed that improvement of external and internal biosecurity, achieved over a period of several months, and better herd management have led breeding farms to reduce antibiotic usage and increase PW (31, 32, 41). This supports the idea that biosecurity should be a core objective of the pig industry. Several studies have shown that improvement in biosecurity, such as by implementing an AIAO system with good cleaning and disinfection, had a beneficial impact on disease control and pig health (26, 31, 42, 43). Moreover, a cost reduction and decline in the percentage of mortality were achieved in another study after implementing biosecurity measures and reducing antibiotic usage (44). The negative correlation between ep, pl, hs, tail, ppa, abscess, and internal biosecurity in this study further highlights the potential importance of a good biosecurity to reduce health issues.

In this study, a higher total biosecurity score was significantly and positively correlated to ADG, PBA, and PW. The level of biosecurity was associated with the number of PW in the study of Postma et al. (31), but not in the study of Backhans et al. (33). Similarly to health indicators, biosecurity was not correlated to the percentage of mortality. A correlation between biosecurity and mortality was found in the study of Maes et al. (45), but not in the most recent study of Laanen et al. (19). Despite a high level of biosecurity in cluster 2, a higher level of mortality was identified. This suggests that the increase of mortality is not only the consequence of infection, but may result from multiple factors.

#### Biosecurity and Welfare

Previous studies showed better welfare when internal biosecurity measures, such as reducing stocking density, have been implemented (46, 47). Moreover, good management and appropriate infrastructures in intensive systems are key elements for better welfare (48), just as for implementing biosecurity measures (19). Although a link of causality cannot be inferred in this study, farms from cluster 1 with low biosecurity and higher level of stl and body marks had lower performance, confirming that poor animal welfare tends to appear in a context of lower biosecurity. Surprisingly, farms from cluster 3 with good biosecurity score had a higher level of pigs requiring hospitalization and lameness. Moreover, higher biosecurity scores were correlated overall to a higher prevalence of pigs requiring hospitalization, suggesting that good management of hospital pens cannot be inferred from a good biosecurity level. Our analysis also showed that an increase in internal biosecurity score was associated with a reduction in prevalence of stl. These observations confirm previous results where a higher biosecurity level was associated with healthier animals and better welfare (31, 32).

### The Interconnections between Health, Welfare, and Performance

The results of correlation coefficients and the PCA were used in combination to understand the interconnection between health, welfare, and performance. Health and welfare indicators used in this study have been described in previous papers (18, 21, 49), and the results for our sample were consistent with these reports. However, several other interconnections between health, welfare, and performance are newly described in the current study.

#### The Interconnections between Health and Welfare

Previous studies have highlighted the connection between different pig pathologies (49) and different welfare outcomes (14, 21). Prevalence of ep and pleuritic lesions were highly correlated in the present dataset; similar risk factors and correlation between ep and pl have been reported in several studies (50–52). However, none of these lung lesions had a strong correlation with the other, less prevalent, health indicators.

Our results showed that a higher level of tail biting could be concomitantly identified by the two different schemes (BPHS, Real Welfare), suggesting a certain accuracy to identify on-farm tail-biting problems in abattoir screening. All welfare outcomes measured on farm were correlated to some of the BPHS lesions, suggesting potential common risk factors or biological connection between health and welfare (49). The prevalence of lameness and stl was associated with the prevalence of EP-like lesions. Previous studies have demonstrated that the prevalence of tail lesions tends to increase the risk of infection leading to acute phase protein elevation and abscesses (15) and lung lesions (16). The prevalence of pyaemia was correlated to the prevalence of stl, but also lameness and sbm. The economic impact of pyaemia has been discussed in the literature and it has been reported as an important cause of condemnation at the slaughterhouse (53). Our study suggests that the prevalence of pyaemia could also be used as a proxy to alert to possible on farm welfare issues; as suggested by Sanchez-Vazquez et al. (49), the presence of one pathology could motivate investigations for other issues.

#### The Interconnections between Health and Performance

The positive correlation identified between FCR and ADG was unexpected. However, the interaction between feed composition and environment on ADG and FCR does not preclude such a relationship (54). The classification of the farms in different clusters and the correlations between variables enabled us to identify some interconnections of EP-like lesions, pleurisy, peritonitis, and tail-biting and lower ADG. A higher prevalence of EP-like lesions and pleurisy has been associated with lower performance in previous studies (11, 12, 55, 56) and confirms the connection between respiratory problems and poor pig performance. However, no correlations were identified in this study between BPHS lesions and mortality, as was also the case in a previous study where antibiotic usage was used as a health indicator (32).

#### The Interconnections between Welfare and Performance

Farms in cluster 1, with the lowest ADG, also tended to have a higher percentage of sbm and tail lesions. This confirms the results of Sinisalo et al. (13), who identified a better ADG for pigs without tail lesions and might explain the connection between lower welfare and economic losses (57). Moreover, a higher prevalence of lameness and pigs requiring hospitalization was correlated to higher mortality. The connection between welfare indicators and production performance is encouraging, as it suggests welfare improvement will not necessarily jeopardize performance. Better performance leading to better economic results has been identified as the main incentive to participate in a quality assurance scheme, while the distrust in economic advantages was the main barrier not to participate in these schemes (58).

## Conclusion

This study highlights the challenges associated with connecting different data sources and conducting relevant analysis for the livestock (pig) industry.

The priority should be given to internal biosecurity, which was generally lower and strongly connected to the general level of biosecurity. While the biosecurity can be improved by taking further measures or adopting new habits, this study also suggests possible limitations in farm infrastructures and a smaller impact of biosecurity regarding issues like mortality, prevalence of lameness and pigs requiring hospitalization.

The interconnections identified between health indicators, welfare outcomes, and production performance appear as a compelling reason to consider the improvement of animal welfare as one of the main objective of the pig industry. Facilitating the data collection and the connections between different sources of information related to biosecurity, health, welfare, and performance would be of importance for the pig industry. This could be beneficial to determine the priority measures that should be adopted to sustain an effective pig production.

## Author Contributions

All the authors have participated to this work, giving several inputs to the methodology and the discussion. The work has been conducted under the PROHEALTH project. All the authors are actively involved in the project.

## Conflict of Interest Statement

The research was conducted in the absence of any commercial or financial relationships that could be construed as a potential conflict of interest.

## Footnotes

^1^http://pork.ahdb.org.uk/health-welfare/health/safe-traceable-pork/bphs/.

^2^http://www.biocheck.ugent.be/biocheck.php
