# Supplementary material for: Connecting Different Data Sources to Assess the Interconnections between Biosecurity, Health, Welfare, and Performance in Commercial Pig Farms in Great Britain
Source: Front Vet Sci. 2018 Mar 6;5:41. doi: 10.3389/fvets.2018.00041 (PMC5845643; doi:10.3389/fvets.2018.00041)
Supplement: Supplementary file 1 [file data_sheet_1.PDF]

Table S1 : Pearson[(*)] or Spearman correlations between biosecurity scores for 40 fattening pig farms visited in GB in 2015-16

	A[1]	B[1*]	C[1*]	D[1]	Es[1]	F[1]	EXT*	G[2]	H[2]	I[2]	J[2]	K[2]	L[2]	INT*	TOT[3]*
A[1]	1														
B[1]	0.27	1													
C[1*]	0.13	0.25	1												
D[1*]	0.04	**0.31**	**0.41**	1											
E[1]	-0.08	0.15	**0.30**	0.26	1										
F[1]	0.04	-0.10	-0.23	0.00	-0.12	1									
EXT*	**0.35**	**0.63**	**0.70**	**0.67**	**0.55**	-0.01	1								
G[2]	0.09	0.17	0.18	0.15	0.15	0.00	0.26	1							
H[2]	0.21	0.07	-0.16	0.13	-0.24	0.21	-0.02	-0.05	1						
I[2]	0.09	0.27	-0.02	**0.31**	0.04	0.10	0.24	0.20	**0.42**	1					
J[2]	0.12	0.18	**0.31**	0.28	0.26	0.12	**0.46**	**0.42**	-0.13	0.11	1				
K[2]	0.25	0.08	**0.33**	**0.43**	**0.30**	0.15	**0.43**	0.22	-0.09	0.12	0.28	1			
L[2]	0.05	0.21	0.28	0.27	**0.38**	-0.10	**0.32**	**0.42**	**-0.31**	0.07	**0.32**	**0.55**	1		
INT*	0.14	0.24	**0.43**	**0.45**	**0.46**	0.08	**0.61**	**0.55**	-0.27	0.13	**0.61**	**0.75**	**0.84**	1	
TOT[3]*	0.20	**0.38**	**0.58**	**0.58**	**0.57**	0.04	**0.82**	**0.48**	-0.24	0.16	**0.62**	**0.74**	**0.73**	**0.95**	1

Significant correlations in bold: moderately correlated if coefficient $r > 0.3$ and $P < 0.05$, strongly correlated if $r > 0.6$ and $P < 0.05$

[1] External biosecurity (EXT) sub-categories scores: A. Purchase of animals and semen; B. Transport of animals, removal of manure/dead animals; C. Feed, water and equipment supply; D. Personnel and visitor; E. Vermin/bird control; F. Environment and region.

[2] Internal biosecurity (INT) sub-categories scores: G. Disease management; H. Farrowing period; I. Nursery, J. Fattening pigs; K. Measures between compartments and the use of equipment; L. Cleaning and disinfection.

[3]Total biosecurity(TOT)= mean (EXT+INT)

Table S2: Pearson[(*)] or Spearman correlations between biosecurity scores for 28 breeding pig farms visited in GB in 2015-16

	A[1]	B[1]	C[1*]	D[1*]	Es[1]	F[1]	EXT*	G[2]	H[2]	I[2]	J[2]	K[2]	L[2]	INT*	TOT[3]*
A[1]	1														
B[1]	0.20	1													
C[1*]	-0.25	-0.11	1												
D[1*]	-0.33	0.03	0.51	1											
E[1]	-0.07	0.27	0.22	0.16	1										
F[1]	0.15	-0.21	**-0.37**	-0.12	**-0.56**	1									
EXT*	0.04	**0.39**	**0.62**	**0.66**	**0.53**	-0.28	1								
G[2]	0.01	-0.05	0.21	**0.31**	0.03	-0.16	0.20	1							
H[2]	0.25	-0.02	-0.27	-0.03	-0.06	0.15	-0.11	-0.20	1						
I[2]	0.08	0.24	0.18	0.29	**0.51**	-0.18	**0.43**	0.28	-0.17	1					
J[2]	**0.35**	0.12	-0.11	-0.11	-0.07	0.26	0.09	0.01	0.00	0.28	1				
K[2]	0.01	-0.12	0.14	0.24	0.04	0.12	0.21	0.25	-0.06	**0.39**	0.18	1			
L[2]	-0.17	0.20	0.09	0.13	0.14	-0.10	0.13	**0.46**	-0.18	0.26	0.17	0.26	1		
INT*	-0.01	0.15	0.24	**0.30**	0.13	0.03	**0.44**	**0.51**	-0.16	**0.63**	**0.48**	**0.63**	**0.72**	1	
TOT[3]*	0.00	0.25	**0.44**	**0.50**	**0.34**	-0.12	**0.75**	**0.47**	-0.14	**0.68**	**0.38**	**0.58**	**0.75**	**0.87**	1

Significant correlations in bold: moderately correlated if coefficient $r > 0.3$ and $P < 0.05$, strongly correlated if $r > 0.6$ and $P < 0.05$.

[1] External biosecurity (EXT) sub-categories scores: A. Purchase of animals and semen; B. Transport of animals, removal of manure/dead animals; C. Feed, water and equipment supply; D. Personnel and visitor; E. Vermin/bird control; F. Environment and region.

[2] Internal biosecurity (INT) sub-categories scores: G. Disease management; H. Farrowing period; I. Nursery, J. Fattening pigs; K. Measures between compartments and the use of equipment; L. Cleaning and disinfection.

[3]Total biosecurity(TOT)= mean (EXT+INT)

Table S3 : Pearson(*) or Spearman correlations between biosecurity scores, health indicators, welfare outcomes and production performance for 40 fattening pig farms for 2015-2016.

	EXT*	INT*	TOT*	**MOR**	**FCR**	**ADG***	hosp	lam	stl	sbm	*ep*	*pl*	*pc*	*pt*
EXT*	1.00													
INT*	**0.61**	1.00												
TOT*	**0.82**	**0.95**	1.00											
**MOR**	-0.09	0.01	-0.01	1.00										
**FCR**	0.19	**0.46**	**0.41**	0.11	1.00									
**ADG***	**0.38**	0.24	**0.32**	**-0.40**	**0.40**	1.00								
hosp	**0.36**	**0.47**	**0.46**	**0.34**	**0.30**	-0.16	1.00							
lam	0.10	-0.02	0.05	**0.67**	-0.20	-0.22	**0.45**	1.00						
stl	-0.04	**-0.31**	-0.18	0.28	-0.14	-0.22	0.18	**0.32**	1.00					
sbm	0.01	-0.26	-0.11	0.08	-0.09	**-0.56**	0.22	0.06	**0.45**	1.00				
ep	0.02	**-0.38**	-0.28	0.06	0.02	-0.16	-0.27	**0.35**	**0.34**	0.07	1.00			
pl	0.01	**-0.51**	**-0.45**	-0.06	-0.04	**-0.30**	-0.19	-0.23	0.20	0.11	**0.66**	1.00		
pc	0.15	-0.19	-0.12	0.15	**0.42**	-0.09	0.28	-0.13	-0.08	0.29	**0.36**	**0.55**	1.00	
pt	-0.02	-0.27	-0.19	-0.25	**-0.58**	-0.04	-0.07	0.12	0.16	**0.45**	0.09	0.06	-0.29	1.00
ms	0.14	0.07	0.15	0.25	**0.39**	0.12	0.11	0.20	-0.16	**0.53**	0.11	-0.04	0.09	**0.36**
hs	**-0.39**	**-0.39**	**-0.39**	0.05	-0.11	0.21	-0.28	0.25	0.08	**0.37**	-0.07	-0.02	-0.04	**0.49**
pd	-0.04	-0.22	-0.18	0.02	-0.24	-0.04	-0.13	0.24	0.28	**0.58**	0.03	0.03	-0.20	**0.64**
tail	-0.29	**-0.50**	**-0.45**	0.17	0.04	-0.21	-0.24	0.15	**0.38**	**0.58**	0.16	0.18	-0.02	**0.37**
viral	0.13	0.18	0.21	0.07	0.16	0.15	0.23	-0.11	-0.23	-0.24	0.06	-0.10	-0.14	0.25
ppa	-0.16	**-0.34**	**-0.35**	-0.21	0.00	0.00	0.10	0.03	-0.06	0.07	**0.32**	**0.52**	0.16	**0.31**
ppc	-0.11	0.11	0.07	**-0.31**	0.27	0.15	0.18	-0.25	0.03	0.18	-0.05	0.12	-0.10	0.03
abscess	-0.08	**-0.34**	-0.27	**-0.33**	-0.02	**0.44**	-0.18	-0.29	0.14	-0.17	-0.05	-0.02	-0.01	**0.42**
pyaemia	-0.06	-0.27	-0.22	0.05	0.16	**0.30**	-0.02	**0.33**	**0.32**	**0.41**	0.08	0.06	0.05	**0.39**
ep score	0.04	-0.16	-0.05	0.00	0.06	0.02	-0.13	**0.52**	0.30	0.15	**0.79**	0.26	0.18	0.23
pl score	-0.11	**-0.54**	**-0.51**	-0.12	-0.15	**-0.36**	-0.13	-0.24	0.19	0.10	**0.51**	**0.90**	**0.33**	0.15
Pd score	-0.05	-0.22	-0.18	0.00	-0.24	-0.04	-0.10	0.21	0.23	**0.57**	0.01	0.04	-0.20	**0.64**

	ms	*hs*	*pd*	*tail*	*viral*	*ppa*	*ppc*	*abscess*	*Pyaemia*	*ep score*	*pl score*	*pd score*
EXT*												
INT*												
TOT*												
**MOR**												
**FCR**												
**ADG***												
hosp												
lam												
stl												
sbm												
ep												
pl												
pc												
pt												
ms	1.00											
hs	**0.39**	1.00										
pd	**0.31**	**0.54**	1.00									
tail	0.24	**0.62**	**0.58**	1.00								
viral	0.27	0.11	-0.11	-0.02	1.00							
ppa	**0.39**	0.27	0.25	0.18	0.09	1.00						
ppc	0.23	0.16	-0.14	-0.01	0.15	**0.42**	1.00					
abscess	0.24	**0.34**	0.11	**0.37**	-0.01	**0.32**	0.21	1.00				
pyaemia	**0.58**	**0.59**	**0.44**	**0.51**	-0.08	**0.51**	**0.34**	**0.62**	1.00			
ep score	0.22	0.07	0.00	0.12	0.26	0.21	-0.02	0.10	0.17	1.00		
pl score	-0.13	-0.02	0.10	0.23	-0.05	**0.57**	0.19	0.11	0.05	0.18	1.00	
Pd score	**0.31**	**0.54**	**1.00**	**0.58**	-0.11	0.27	-0.13	0.11	0.44	-0.01	0.11	1.00

EXT: External biosecurity, INT: internal biosecurity score, TOT: total biosecurity score, **ADG**: Average daily weight gain, **FCR**: Feed conversion ratio, **MOR**: Mortality, hosp: pigs requiring hospitalization, lam: lameness, stl: severe tail lesions, sbm: severe body marks, *ep*: enzootic pneumonia, *pl*: pleurisy, *pc*: pericarditis, *pt*: peritonitis, *ms*: milk spot, *hs*: hepatic scarring, *pd*: papular dermatitis, *tail*: tail-bitten, *viral*: viral-type distribution, *ppa*: pleuropneumonia – acute, *ppc*: pleuropneumonia – chronic, *abscess:* abscess, *pyaemia*: pyaemia, *ep score:* score enzootic pneumonia, *pl score*: score pleurisy, *pd score*: score papular dermatitis

Table S4 : Pearson(*) or Spearman correlations between production performance and biosecurity scores for 28 breeding pig farms for 2015-2016.

	EXT*	INT*	TOT*	**PB***	**PBA***	**PW***
EXT*	1					
INT*	**0.44**	1				
TOT*	**0.75**	**0.87**	1			
**PB***	0.11	**0.33**	0.29	1		
**PBA***	0.15	0.4	**0.36**	**0.93**	1	
**PW***	0.28	**0.43**	**0.44**	**0.73**	**0.86**	1

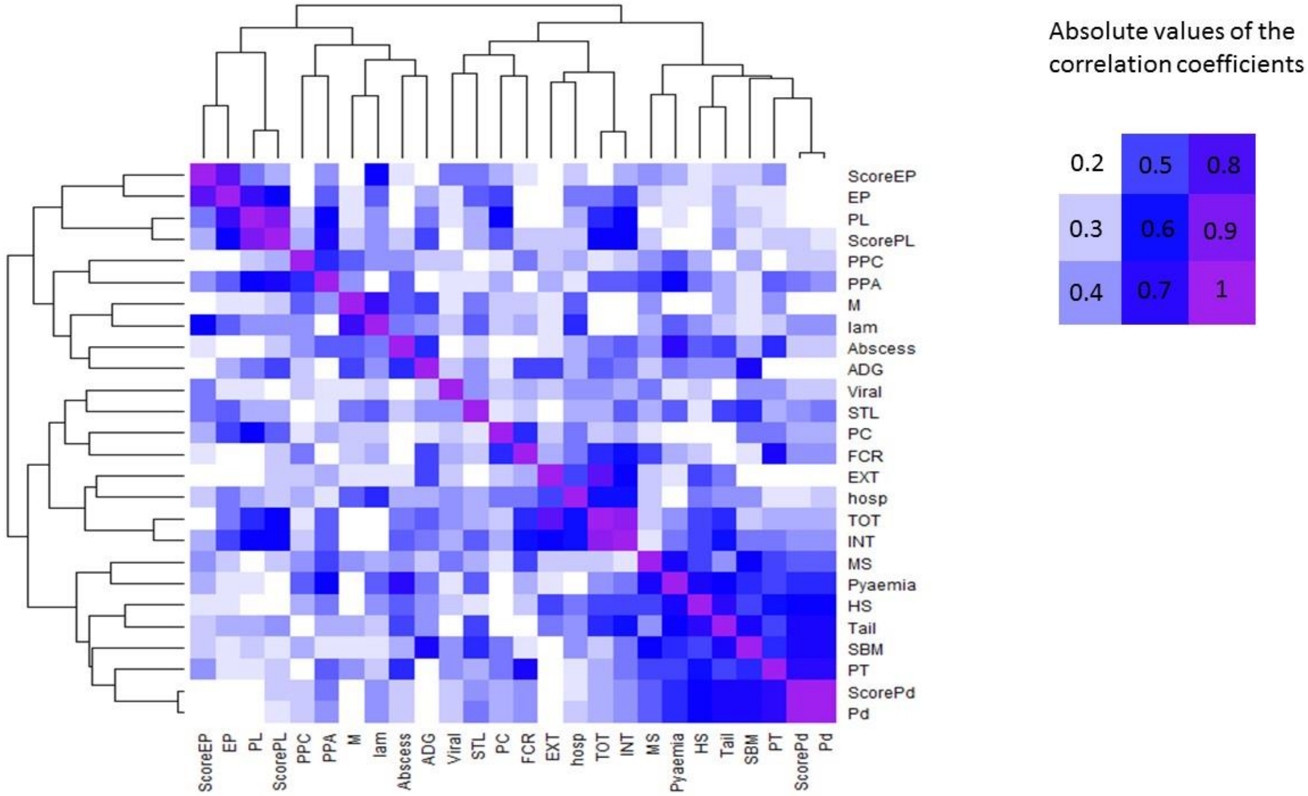

Figure S1 : Correlation matrix: Correlations between welfare outcomes, production performance, health indicators and biosecurity scores for 40 fattening pig farms for 2015-2016. The more intense is the blue colour, the stronger is the correlation between two variables

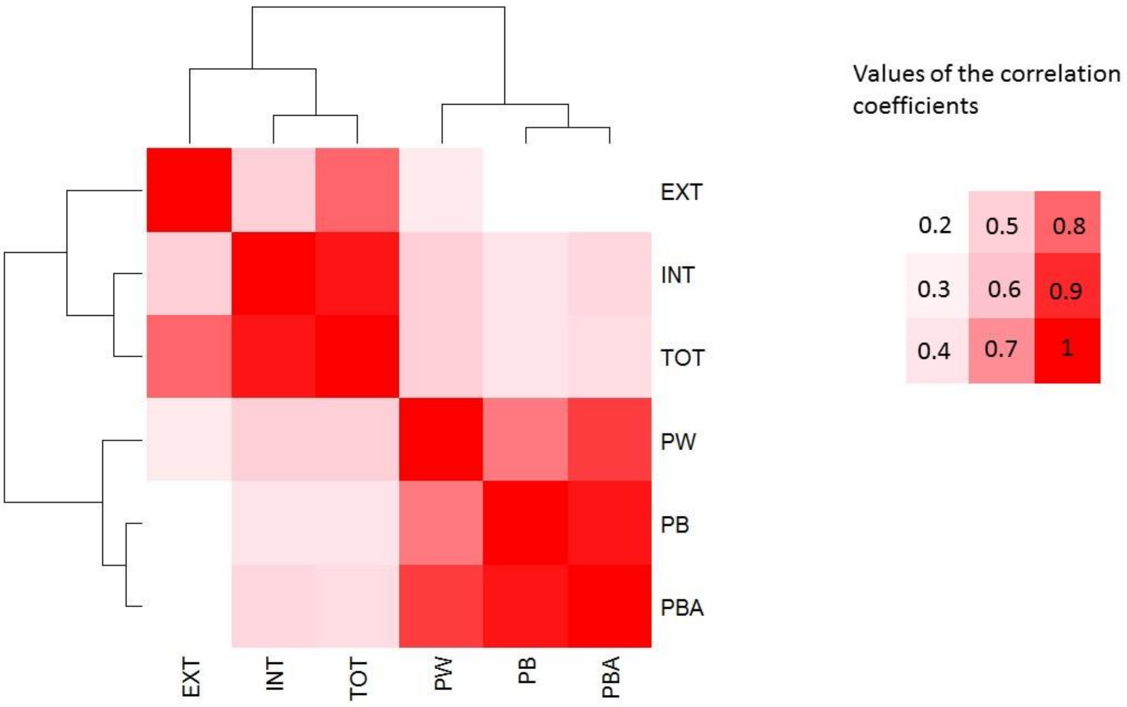

Figure S2 : Correlation matrix: Correlations between production performance and biosecurity scores for 28 breeding pig farms for 2015-2016. The more intense is the red colour, the stronger is the correlation between two variables
